# Case Report: ^18^F-FDG PET-CT for Diagnosing Prosthetic Device-Related Infection in an Infant With CHD

**DOI:** 10.3389/fped.2021.584741

**Published:** 2021-03-08

**Authors:** Junpei Kawamura, Kentaro Ueno, Eri Taimura, Tomoyuki Matsuba, Yutaka Imoto, Megumi Jinguji, Yoshifumi Kawano

**Affiliations:** ^1^Department of Pediatrics, Kagoshima University, Kagoshima, Japan; ^2^Department of Cardiovascular Surgery, Kagoshima University, Kagoshima, Japan; ^3^Department of Radiology, Kagoshima University, Kagoshima, Japan

**Keywords:** PET, congenital heart disease, device infection, *Pseudomonas aeruginosa*, infant, pediatric, SUV max, pseudoaneurysm

## Abstract

Patients who have undergone cardiac surgery using prosthetic devices have an increased risk of developing prosthetic device-related infection and mediastinitis. However, accurate diagnosis of prosthetic device-related infection can be difficult to evaluate and treat with antibiotic therapy alone. In recent years, ^18^F-fluorodeoxyglucose positron emission tomography-computed tomography (^18^F-FDG PET-CT) has made promising contributions to detect infective endocarditis, pacemaker infections, or other inflammations. Nevertheless, ^18^F-FDG PET-CT for congenital heart disease (CHD) with device infection has been sparsely reported. We present an infantile girl diagnosed with pulmonary atresia with a ventricular septal defect who underwent replacement of the right ventricle-to-pulmonary artery (RV-PA) conduit for improvement cyanosis. She developed high fever and was diagnosed with mediastinitis and bacteremia by *Pseudomonas aeruginosa (P. aeruginosa)* on postoperative day 4. Mediastinal drainage and 6 weeks of antibiotic therapy improved her condition, but bacteremia flared up on postoperative day 56. Despite a long course of antibiotic therapy, she had two more recurrences of bacteremia with the detection of *P. aeruginosa*. Echocardiography and chest contrast CT showed no evidence of vegetation and mediastinitis. On postoperative day 115, ^18^F-FDG PET-CT revealed an accumulation on the RV-PA conduit (SUV max 3.4). Finally, she developed an infectious ventricular pseudo-aneurysm on postoperative day 129 and underwent aneurysm removal and RV-PA conduit replacement on postoperative day 136. Our case showed the importance of ^18^F-FDG PET-CT for diagnosing specific localization of prosthetic device-related infection which is hard to detect using other imaging techniques. It can be a useful diagnostic tool for infantile patients with CHD with cardiac prosthetic devices and improve subsequent clinical treatments.

## Introduction

After pediatric cardiac surgery, 0.25–5.0% of patients develop mediastinitis (1). Negative pressure wound therapy (NPWT) and antibiotic therapy is a reliable option in mediastinitis (2); however, recurrent infections happen even after appropriate treatment. Prosthetic valves and grafts are also significant risk factors for mediastinitis and appropriate attention needs to be paid to this (3). The treatment principle of prosthetic device-related infection requires complete removal of the prosthetic device with concomitant antibiotic therapy. It is difficult to decide whether to remove an infected device surgically when device infection cannot be proven because surgery is invasive for infants, especially in patients with cyanotic heart disease. Recently, the usefulness of ^18^F-fluorodeoxyglucose positron emission tomography-computed tomography (^18^F-FDG PET-CT) to evaluate the presence or absence of prosthetic device-related infection has been reported (4–7), and could be an important tool in determining treatment. Nevertheless, there are only a few reports of ^18^F-FDG PET-CT for prosthetic device-related infection in pediatric patients after cardiac surgery for congenital heart disease (CHD) (8, 9). Understanding the difference between children and adults in the normal distribution and physiologic variants of ^18^F-FDG PET-CT uptake is important to avoid misinterpretation (10). Herein, we present the first infantile cyanotic CHD of recurrent bacteremia and infectious ventricular pseudo-aneurysm due to prosthetic device-related infection. ^18^F-FDG PET-CT findings provided complementary information on prosthetic device-related infection that was crucial for making decisions regarding course of treatment.

## Case Presentation

**Figure 1:**
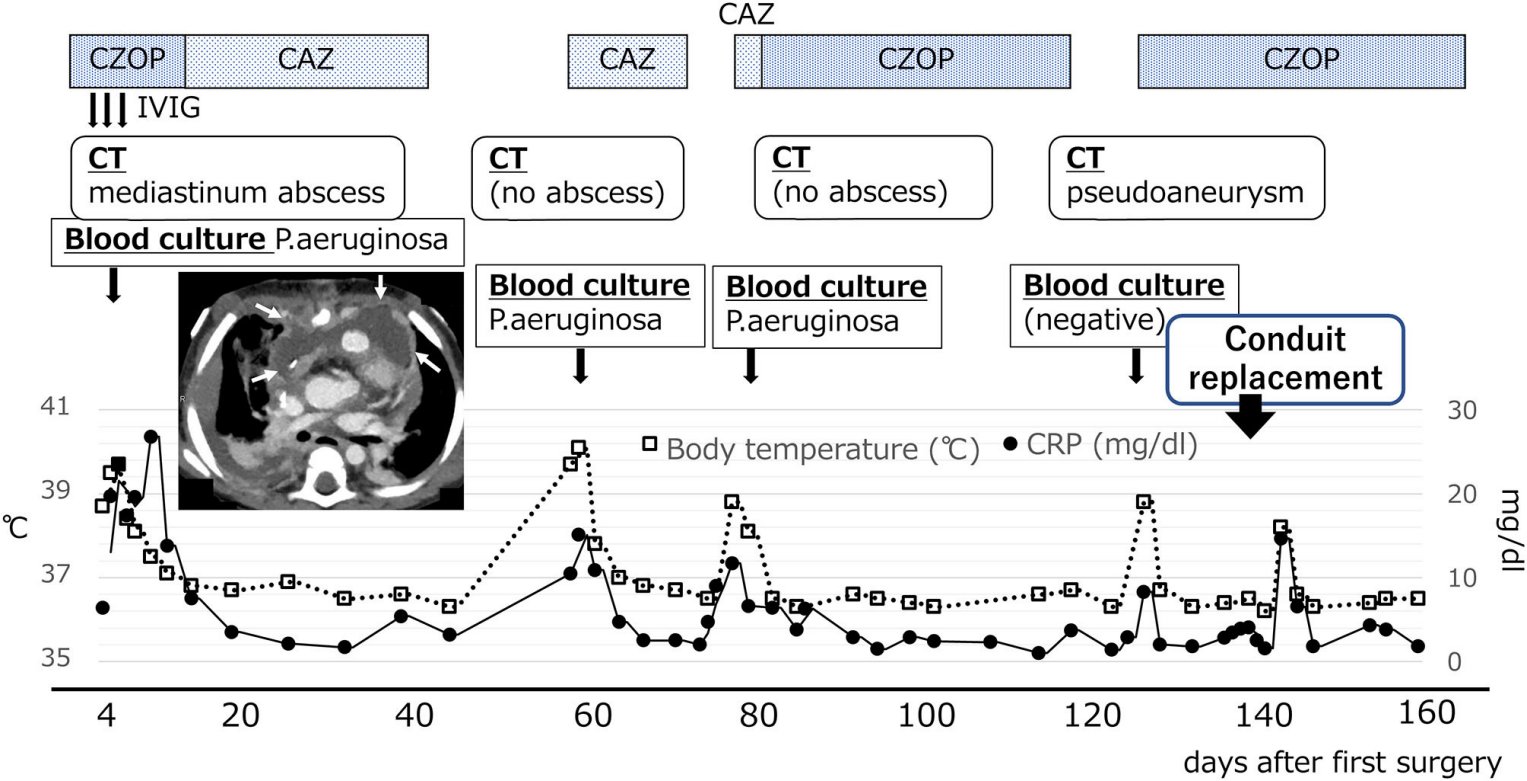
Changes in postoperative temperature and CRP values. Chest CT scan at 7 days after surgery showed a wide low-density area in the front of the RV-PA conduit in the anterior mediastinum (white arrow). CAZ, ceftazidime; CZOP, cefozopran; IVIG, intravenous immunoglobulin G.

A 1-year-old female patient diagnosed with pulmonary atresia with a ventricular septal defect (PA/VSD) had a systemic-to-pulmonary shunt inserted at 25 days of age. Although her SpO_2_ was 75% at discharge, her hypoxemia worsened, and SpO_2_ deteriorated to 60–70% at 8 months of age. At 10 months of age, she underwent a right ventricle-to-pulmonary artery (RV-PA) conduit insertion (10 mm vascular prosthesis with an artificial valve) with resection of the systemic-to-pulmonary shunt. Her cyanosis improved with baseline peripheral oxygen saturation elevated from 70 to 90%, but her condition worsened with persistent high fever (39–40°C). On the 4th postoperative day, a blood culture showed positive results for *Pseudomonas Aeruginosa (P. aeruginosa)*. Chest computed tomography (CT) showed an extensive abscess in the anterior mediastinum (Figure 1), and *P. aeruginosa* was also detected in the culture of the wound pus. The patient was thus diagnosed with mediastinitis and underwent NPWT and mediastinal drainage for 28 days. Intravenous cefozopran was started as the initial antibiotic therapy, which was then revised to intravenous ceftazidime for 6 weeks, based on the antibiotic susceptibility test. Thereafter, two consecutive blood culture tests showed negative results for *P. aeruginosa*, and she was finally discharged on the 46th postoperative day.

**Figure 2:**
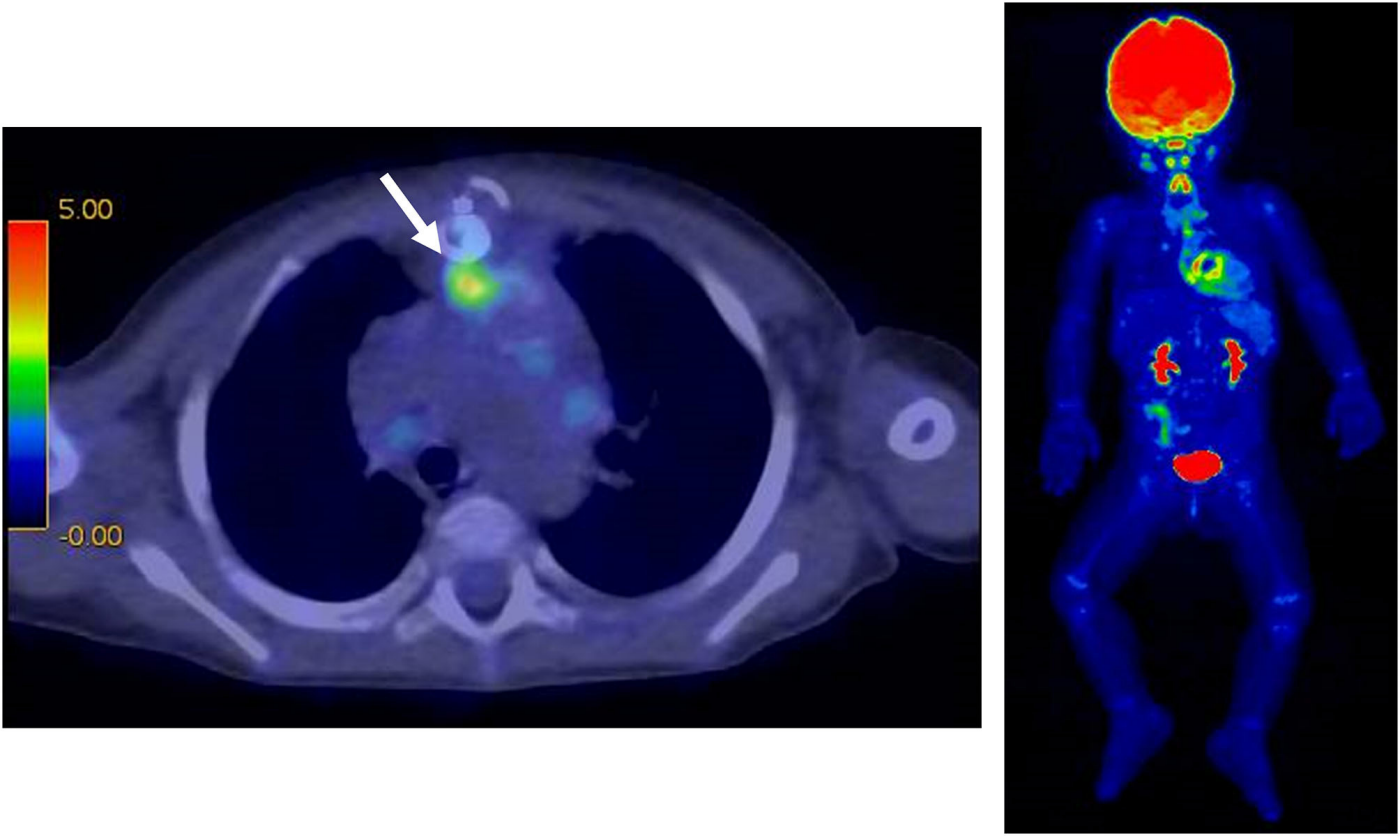
^18^F-FDG PET-CT at 115 days after surgery. There was a mild accumulation (SUV max 3.4) on the RV-PA conduit in the anterior mediastinum (white arrow).

**Figure 3:**
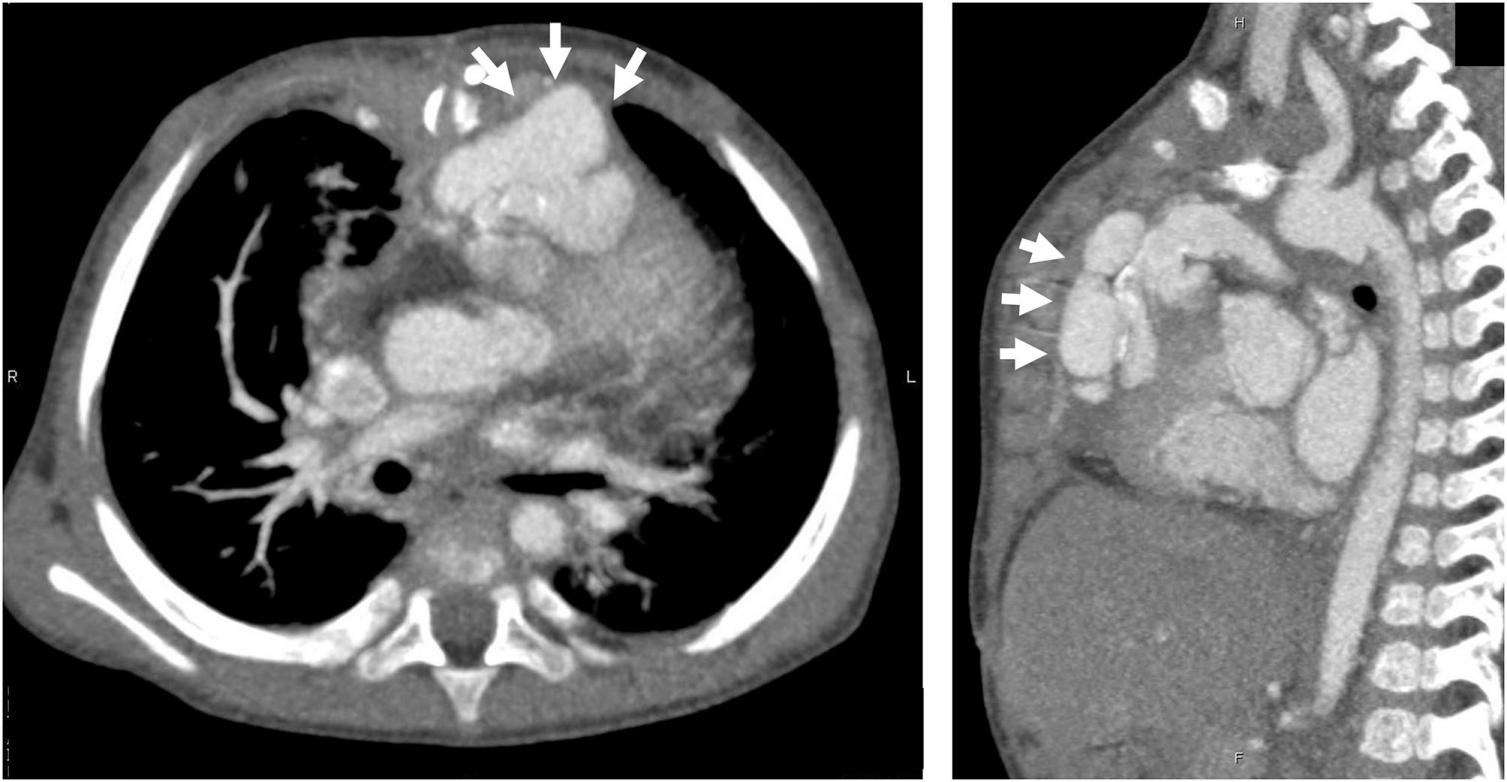
Chest CT scan at 129 days after surgery. There was a ventricular pseudoaneurysm extending from the right ventricular free wall to the posterior surface of the sternum in the mediastinum (white arrow).

However, the patient was readmitted to the hospital on the 57th postoperative day due to high fever (over 39°C) and worsening general condition. At this presentation, her height was 69.5 cm, body weight was 10.5 kg, body temperature was 40.0°C, and there was no middle thoracic wound. Peripheral blood test showed the following results: white blood cell count, 23,540/μL and C-reactive protein level, 15.08 mg/dL. Blood culture was positive for *P. aeruginosa* (minimum inhibitory concentration [MIC] of ceftazidime: 4.0 μg/mL). PCR-based open reading frame typing (POT) confirmed that all the *P. aeruginosa* detected in blood cultures on postoperative days 4, 57, and 77 were of the same genotype: protein of telomeres (POT)1 119, POT2 20, and negative for *blaVIM* and *blaIMP*. Transthoracic echocardiography showed no vegetation and pericardial effusion. There was no valve regurgitation in color doppler imaging. Chest CT showed no abscess in the mediastinum. She was treated with ceftazidime for *P. aeruginosa* bacteremia for 2 weeks but had recurrent fever on the 77th postoperative day. Two blood culture tests showed *P. aeruginosa* positivity, and the MIC of ceftazidime was elevated to 8.0 μg/mL, whereas the MIC of cefozopran was <2.0 μg/mL. Hence, antibiotic therapy was switched to cefozopran on the 79th postoperative day. Chest contrast CT on the 86th postoperative day did not show an intra-mediastinal abscess. She was then diagnosed with infective endocarditis (IE) according to the Duke classification and treated with cefozopran for a total duration of 6 weeks. On the 115th postoperative day, ^18^F-FDG PET-CT-administered 28 MBq in removal of the carbohydrate showed mild accumulation (SUV max 3.4, SUV ratio 3.2) at the RV-PA conduit (Figure 2). On the 123rd postoperative day, she had recurrent high fever, and cefozopran administration was resumed. On the 129th postoperative day, her SpO_2_ dropped below 75%, and contrast CT of the chest revealed a ventricular aneurysm adjacent to and compressing the RV-PA conduit (Figure 3). As a result, she underwent pseudoaneurysm removal and RV-PA conduit replacement on the 136th postoperative day. Cultures of previous pericardial sheets and RV-PA conduit submitted intraoperatively showed the presence of *P. aeruginosa*. After this surgery, her hypoxemia improved, and she was treated with NPWT for an additional 8 days and cefozopran for the total duration during the treatment course. Currently, the patient is doing well and has had no recurrent fever.

## Discussion

Acute infectious mediastinitis is a serious complication that occurs after cardiac surgery and has a mortality rate of 7–40% (11). Treatment usually involves 3–6 weeks of antibiotic therapy. Additionally, surgical mediastinal drainage and NPWT have been recommended (2, 12). Nevertheless, more than 20% of cases have recurrent mediastinitis (13). The recurrence rate of mediastinitis associated with IE or prosthetic device-related infection is high, and patients have a poor prognosis when treated with pharmacotherapy alone (14, 15). The sensitivity of CT to diagnose mediastinitis is not high (67%) (16), and only 18–57% patients with mediastinitis were blood-culture positive (17). On the other hand, surgical intervention for IE associated with RV-PA conduits is associated with a mortality of 17% (18). Therefore, in patients with bacteremia who are successfully treated with antibiotic therapy, it is difficult to decide whether surgical removal of the prosthetic device is warranted when the presence of mediastinitis and prosthetic device-related infection cannot be identified by contrast-enhanced CT imaging. Transesophageal echocardiography (TEE) is useful for detecting lesions of IE even when no abnormality is seen on TTE (19). However, in our case, no perivalvular mass or color doppler abnormality was seen on TTE, in addition, we suspected the anterior prosthetic graft as the source of infection before PET-CT scan and it was considered difficult to detect the lesion with TEE observed from the posterior. That was why we did not conducted TEE in this case. ^99m^Tc-HMPAO-labeled leukocyte SPECT/CT has been reported to have a sensitivity of 90% and a specificity of 100% for detecting IE (20). However, leukocyte-labeled SPECT/CT cannot be performed in our hospital due to limited facilities in Japan. Regarding postoperative cardiac surgery in adolescents and adults, ^18^F-FDG PET-CT has been useful to identify pacemaker lead infection, prosthetic valve infection, and graft infection (5–9). ^18^F-FDG PET-CT has a high sensitivity and specificity to diagnose prosthetic vascular graft infection is high (100 and 71.4%, respectively) (21), and might be helpful to evaluate good long-term prognosis and patient survival in the case of vascular graft infection (18). The ESC guidelines for IE include PET-CT findings as one of the major criteria in the Duke classification for prosthetic valve infection (22). Previous reports have shown radiotracer accumulation in the case of device-related infection even 20–30 days after the initiation of antibiotic therapy (23, 24). Therefore, device-related infection may be detected even after the initiation of antibiotic therapy. Herein, we detected abnormal ^18^F-FDG accumulation more than 4 weeks after the initiation of antibiotic therapy, which suggested that the patient was less responsive to pharmacotherapy for the prosthetic valve-related infection. Nevertheless, we believe that the PET-CT findings could confirm the presence of the device-related infection. However, the indication for surgery had to be carefully considered because infants with cyanotic congenital heart disease have a high risk of postoperative mortality with device insertion. Although some cases of ventricular pseudo-aneurysm occurring after IE have been reported (25, 26), our patient developed a pseudo-ventricular aneurysm because of an intra-mediastinal prosthetic device-related infection. Ventricular pseudo-aneurysm is a life-threatening disease, with approximately a 30–45% risk of aneurysm rupture (27). Therefore, we performed immediate prosthetic device replacement, even though ^18^F-FDG PET-CT findings showed mild accumulation.

Moreover, gram-negative bacterial (GNB) mediastinitis has a poor prognosis (11). In this case, the results of culture tests showed persistent and recurrent infections, and elevated MICs of *P. aeruginosa* against ceftazidime (2.0–8.0). In cases of postoperative mediastinitis, *P. aeruginosa* was the least common etiological agent (5.8%) (28). *P. aeruginosa* forms antibiotic-resistant biofilms on a wide range of substrates relevant to human infection (29). In this case, the bacteria formed biofilms according to the same genotypes of each bacteria. Prolonged and repeated antibiotic therapy might contribute to the development of antibiotic resistance.

There is no clear criterion for classifying ^18^F-FDG PET-CT positivity in pediatric cases of postoperative device-related infections with congenital heart disease, and only a few reports are available in the literature on postoperative device infections in patients with CHD (Table 1). This is the first infantile CHD case with intra-mediastinal prosthetic graft-related infection, which was identified by ^18^F-FDG PET-CT. An SUV max of 3.485 or higher as a threshold for ^18^F-FDG PET-CT can diagnose pacemaker device infection with a sensitivity of 91.3% and specificity of 93.7% (26). An SUV max of 5.6 or higher could diagnose aortic stent graft infection with a sensitivity of 89% and specificity of 100% (31). An SUV max value is generally weight-corrected. Nevertheless, in a review of pediatric patients aged 2–17 years and weighing 11–77 kg, SUV max values in normal liver and tumors (range, 1.3–13.7; median, 3.4) were positively correlated with weight (32), which indicates that infants have a lower weight-corrected SUV max than adolescents and school-aged children. A previous report of rhabdomyosarcoma showed that the SUV max of a 1-year-old female child was 3.3 with a malignant lesion, which was lower than that observed for other children (33). Other pediatric studies have shown that the SUV max of arthritic sites was 2.0 ± 0.6 in 5.4 ± 4.0-year-old juvenile patients with idiopathic arthritis (34). Most importantly, an SUV ratio of 2.0 was most sensitive and specific for prosthetic heart valve endocarditis in adults (35). The SUV ratio in this case was calculated by dividing the SUV max around the prosthetic device by the mean SUV in the blood pool (aortic arch): 3.08/0.96=3.2. All these studies indicate that the SUV observed in our patient was not low but significant for the diagnosis of prosthetic graft-related infection. However, a time period of ~3 months is said to be sufficient for the healing of inflammatory changes and resolution of FDG uptake (36). Technical precautions are needed to avoid false positives such as adequate suppression of myocardial FDG uptake when detecting heart infection, so we need to be careful in our interpretation of the SUV value when detecting infantile prosthetic device-related infection or IE.

**Table 1:** Prosthetic device infection cases diagnosed by FDG-PET-CT in CHD (congenital heart disease).

**No.**	**Investigator**	**Age (y)**	**Sex**	**CHD**	**Position**	**SUV max**	**Bacteria**	**Initial TTE**	**Therapy**	**Surgery**
1	Roque et al. (5)	48	M	TOF	Bioprosthetic pulmonary valve	8.04	Unknown	Negative	Unknown	Unknown
2	Zhang et al. (7)	9	F	TOF	Pulmonary conduit	4.2	MRSA	Positive	Antibiotic	Conduit replacement
3	Meyer et al. (30)	11	M	TOF	Pulmonary conduit valve	Unknown	Staphylococcus aureus	Positive	Antibiotic	None
4	Meyer et al. (30)	55	F	TOF	Pulmonary conduit valve	Unknown	Staphylococcus aureus	Negative	Antibiotics	Conduit replacement
5	Meyer et al. (30)	17	F	TOF	Pulmonary conduit valve	Unknown	Staphylococcus capitis	Negative	Antibiotics	None
6	Meyer et al. (30)	8	F	TAC	Pulmonary conduit	Unknown	Lactobazillus rhamnosus	Negative	Antibiotics	None
7	Meyer et al. (30)	22	F	TOF	Pulmonary conduit	Unknown	Negative	Negative	Antibiotics	None

*TTE, transesophageal echocardiography; TOF, tetralogy of Fallot; TAC, Truncus arteriosus; MRSA, methicillin resistant Staphylococcus aureus*.

Our study is a single case report, therefore there are some limitations. Though ^18^FDG PET-CT is a promising tool to empower the sensibility and specificity of simple CT scans and has even been cited in the latest IE ESC guidelines (2015) (23), it is still difficult to determine whether the SUV max and SUV ratio threshold can fit in the IE cases especially if the patients are infants. After open-heart surgery, they are greatly affected by the number of days postoperatively. In the wide variety of operations for congenital heart disease, there may be differences in the degree of accumulation of PET-CT during infection. Further research is needed to examine the criteria for diagnosing prosthetic device-related infection in infants using ^18^F-FDG PET-CT.

## Conclusion

^18^F-FDG PET-CT can be a useful modality to diagnose device-related infections in infants and to devise an optimal treatment plan. It can also ascertain whether device removal and mediastinal drainage is necessary in patients with CHD, even in pediatric patients.

## Data Availability Statement

The original contributions presented in the study are included in the article/supplementary material, further inquiries can be directed to the corresponding author/s.

## Ethics Statement

Written informed consent was obtained from the minor's legal guardian/next of kin for the publication of any potentially identifiable images or data included in this article.

## Author Contributions

JK and KU conceptualized and designed the study, drafted the initial manuscript, and reviewed and revised the manuscript. YK, ET, and MJ designed the data collection instruments, collected data, carried out the initial analyses, reviewed and revised the manuscript, and provided critical revision and approval of the article. TM and YI performed the patient's surgery, contributed to the analysis and interpretation of data and revision of the manuscript for important intellectual content. All authors approved the final manuscript as submitted and agree to be accountable for all aspects of the work.

## Conflict of Interest

The authors declare that the research was conducted in the absence of any commercial or financial relationships that could be construed as a potential conflict of interest.

## References

[1] Abu-Omar Y Kocher GJ Bosco P Barbero C Waller D Gudbjartsson T. European association for cardio-thoracic surgery expert consensus statement on the prevention and management of mediastinitis. Eur J Cardiothorac Surg. (2017) 51:10–29. 10.1093/ejcts/ezw326 28077503

[2] Durandy Y. Mediastinitis in pediatric cardiac surgery: prevention, diagnosis and treatment. World J Cardiol. (2010) 2:391–8. 10.4330/wjc.v2.i11.391 21179306 PMC3006475

[3] Yuan SM. Right-sided infective endocarditis: recent epidemiologic changes. Int J Clin Exp Med. (2014) 7:199–218. 24482708 PMC3902260

[4] Sarrazin JF Philippon F Tessier M Guimond J Molin F Champagne J. Usefullness of fluorine-18 positron emission tomography/computed tomography for identification of cardiovascular implantable electronic device infections. J Am Coll Cardiol. (2012) 59:1616–25. 10.1016/j.jacc.2011.11.059 22538331

[5] Roque A Pizzi MN Cuéllar-Calàbria H Aguadé-Bruix S. ^18^F-FDG-PET/CT angiography for the diagnosis of infective endocarditis. Curr Cardiol Rep. (2017) 19:15. 10.1007/s11886-017-0824-3 28185172

[6] Chen W Sajadi MM Dilsizian V. Merits of FDG PET/CT and functional molecular imaging over anatomic imaging with echocardiography and CT angiography for the diagnosis of cardiac device infections. JACC Cardiovasc Imaging. (2018) 11:1679–91. 10.1016/j.jcmg.2018.08.026 30409329

[7] Swart LE Scholtens AM Tanis W Nieman K Bogers AJJC Verzijlbergen FJ. ^18^F-fluorodeoxyglucose positron emission/computed tomography and computed tomography angiography in prosthetic heart valve endocarditis: from guidelines to clinical practice. Eur Heart J. (2018). 39:3739–49. 10.1093/eurheartj/ehx784 29351615

[8] Zhang Y Williams H Pucar D. FDG-PET identification of infected pulmonary artery conduit following tetralogy of fallot (TOF) repair. Nucl Med Mol Imaging. (2017) 51:86–7. 10.1007/s13139-016-0424-y 28250862 PMC5313460

[9] Morishima S Ono T Honda M Kanno M Midorikawa H Ishikawa K. Detection of late presentation of poststernotomy mediastinitis in an infant by positron emission tomography. Jpn. J. Cardiovasc. Surg. (2008) 37:96–9. 10.4326/jjcvs.37.96

[10] Shammas A Lim R Charron M. Pediatric FDG PET/CT: physiologic uptake, normal variants, and benign conditions. Radiographics. (2009) 29:1467–86. 10.1148/rg.295085247 19755606

[11] Charbonneau H Maillet J M. Faron M Mangin O Puymirat E. Mediastinitis due to gram-negative bacteria is associated with increased mortality. Clin Microbiol Infect. (2014) 20:197–202. 10.1111/1469-0691.12369 24520879

[12] Anslot C Hulin S Durandy Y. Postoperative mediastinitis in children: improvement of simple primary closed drainage. Ann Thorac Surg. (2007) 84:423–8. 10.1016/j.athoracsur.2007.03.064 17643610

[13] McNeil JC Campbell JR Crews JD. Healthcare-Associated Infections in Children. A Guide to Prevention and Management., Houston, TX: Springer (2019). p. 180. 10.1007/978-3-319-98122-2

[14] Bisoyi S Dash AK Nayak D Sahoo S Mohapatra R. Left ventricular pseudoaneurysm versus aneurysm a diagnosis dilemma. Ann Card Anaesth. (2016) 19:169–72. 10.4103/0971-9784.173042 26750696 PMC4900369

[15] Rundström H Kennergren C Andersson R Alestig K Hogevik H. Pacemaker endocarditis during 18 years in Göteborg. Scand J Infect Dis. (2004) 36:674–9. 10.1080/00365540410022611 15370655

[16] Baddour LM Bettmann MA Bolger AF Epstein AE Ferrieri P Gerber MA. Nonvalvular cardiovascular device-related infections. Circulation. (2003) 108:2015–31. 10.1161/01.CIR.0000093201.57771.47 14568887

[17] Yamaguchi H Yamauchi H Yamada T Ariyoshi T Aikawa H Kato Y. Diagnostic validity of computed tomography for mediastinitis after cardiac surgery. Ann Thorac Cardiovasc Surg. (2001) 7:94–98. 11371278

[18] Yusuf E Chan M Renz N Trampuz A. Current perspectives on diagnosis and management of sternal wound infections. Infect Drug Resist. (2018) 11:961–8. 10.2147/IDR.S130172 30038509 PMC6053175

[19] Vilacosta I Graupner C San Román JA Sarriá C Ronderos R Fernández C. Risk of embolization after institution of antibiotic therapy for infective endocarditis. J Am Coll Cardiol. (2002) 39:1489–95. 10.1016/S0735-1097(02)01790-4 11985912

[20] Erba PA Conti U Lazzeri E Sollini M Doria R De Tommasiet SM. Added value of 99mTc-HM- PAO-labeled leukocyte SPECT/CT in the characterization and management of patients with infectious endocarditis. J Nucl Med. (2012) 53:1235–43. 10.2967/jnumed.111.099424 22787109

[21] Maria Bonou Chris J Kapelios Michael Samarkos George Benetos Maria Tampaki Nikoletta Pianou. Early diagnosis of right ventricle-pulmonary artery conduit endocarditis by PET/CT. Int J Infect Dis. (2018) 68:24–5. 10.1016/j.ijid.2017.12.020 29277317

[22] Karaca S Rager O Ratib O Kalangos A. Long-term results confirmed that 18F-FDG-PET/CT was an excellent diagnostic modality for early detection of vascular grafts infection. Q J Nucl Med Mol Imaging. (2018) 62:200–8. 10.23736/1824-4785.16.02746-1 25319041

[23] Habib G Lancellotti P Antunes MJ Bongiorni MG Casalta JP Del Zotti F. 2015 ESC Guidelines for the management of infective endocarditis: The Task Force for the Management of Infective Endocarditis of the European Society of Cardiology (ESC). Endorsed by: European Association for Cardio-Thoracic Surgery (EACTS), the European Association of Nuclear Medicine (EANM). Eur Heart J. (2015) 36:3075–128. 10.1093/eurheartj/ehv319 26320109

[24] Granados U Fuster D Pericas JM Llopis JL Ninot S Quintana E. Diagnostic accuracy of 18F-FDG PET/CT in infective endocarditis and implantable cardiac electronic device infection: a cross-sectional study. J Nucl Med. (2016) 57:1726–32. 10.2967/jnumed.116.173690 27261514

[25] De Feo M De Santo LS Romano G Renzulli A Corte AS Utili R. Treatment of recurrent staphylococcal mediastinitis: still a controversial issue. Ann Thorac Surg. (2003) 75:538–42. 10.1016/S0003-4975(02)04313-8 12607669

[26] Arora K Das RR Tandon R Goyal K Panda SS. Pseudoaneurysm of left ventricle following staphylococcal pericarditis in a child. APSP J Case Rep. (2015) 6:26. 26623253 PMC4648146

[27] Sykes MC Nathan M Sanders SP J Gauvreau K Pigula FA Rhodes J. Pseudoaneurysm complicating right ventricle-to-pulmonary artery conduit surgery: incidence and risk factors. J Thorac Cardiovasc Surg. (2017) 154:2046–9. 10.1016/j.jtcvs.2017.08.014 28919137

[28] Zogala D Rucka D Ptacnik V Cerny V Trnka J Varejka P. How to recognize stent graft infection after endovascular aortic repair: the utility of 18F-FDG PET/CT in an infrequent but serious clinical setting. Ann Nucl Med. (2019) 33:594–605. 10.1007/s12149-019-01370-9 31144118

[29] Orvin K Goldberg E Bernstine H Groshar D Sagie A Kornowski R. The role of FDG-PET/CT imaging in early detection of extra-cardiac complications of infective endocarditis. Clin Microbiol Infect. (2015) 21:69–76. 10.1016/j.cmi.2014.08.012 25636930

[30] Zora Meyer M Fischer J Koerfer K T Laser D Kececioglu W Burchert. The role of FDG-PET-CT in pediatric cardiac patients and patients with congenital heart defects. Int J Cardiol. (2016) 220:656–60. 10.1016/j.ijcard.2016.06.109 27393845

[31] Mah TF O'Toole GA. Mechanisms of biofilm resistance to antimicrobial agents. Trends Microbiol. (2001) 9:34–9. 10.1016/S0966-842X(00)01913-2 11166241

[32] Yeung HW Sanches A Squire OD Macapinlac HA Larson SM Erdi YE. Standardized uptake value in pediatric patients: an investigation to determine the optimum measurement parameter. Eur J Nucl Med Mol Imaging. (2002) 29:61–6. 10.1007/s00259-001-0662-8 11807608

[33] Shan ZY Leiker AJ Onar-Thomas A Li Y Feng T Reddick WE. Cerebral glucose metabolism on positron emission tomography of children. Hum Brain Mapp. (2014) 35:2297–309. 10.1002/hbm.22328 23897639 PMC4084709

[34] Tateishi U Imagawa T Kanezawa N Okabe T Shizukuishi K Inoue T. PET assessment of disease activity in children with juvenile idiopathic arthritis. Pediatr Radiol. (2010) 40:1781–8. 10.1007/s00247-010-1716-5 20523983

[35] Swart LE Gomes A Scholtens AM Sinha B Tanis W Lam MGEH. Improving the diagnostic performance of 18 f-fluorodeoxyglucose positron-emission tomography/computed tomography in prosthetic heart valve endocarditis. Circulation. (2018) 138:1412–27. 10.1161/CIRCULATIONAHA.118.035032 30018167

[36] Garg G Benchekroun MT Abraham T. FDG-PET/CT in the postoperative period: utility, expected findings, complications, and pitfalls. Semin Nucl Med. (2017) 47:579–94. 10.1053/j.semnuclmed.2017.07.005 28969758

